# *N*-(4-Chloro­butano­yl)-*N*′-phenyl­thio­urea

**DOI:** 10.1107/S1600536811001498

**Published:** 2011-01-15

**Authors:** Bohari M. Yamin, Nur Eliyanti Ali Othman, M. Sukeri M. Yusof, Farhana. Embong

**Affiliations:** a School of Chemical Sciences and Food Technology, Universiti Kebangsaan Malaysia, UKM 43600 Bangi Selangor, Malaysia; b Department of Chemical Sciences, Faculty of Science and Technology, Universiti Malaysia Terengganu, Menggabang Telipot, 21030 Kuala Terengganu, Malaysia

## Abstract

The asymmetric unit of the title compound, C_11_H_13_ClN_2_OS, contains two independent mol­ecules. Both mol­ecules maintain a *trans*–*cis* configuration with respect to the position of the carbonyl group and the benzene ring against the thione group across the C—N bonds. The mol­ecules are stabilized by intra­molecular N—H⋯O hydrogen bonds. In the crystal, the mol­ecules are linked by inter­molecular N—H⋯S, N—H⋯O and C—H⋯S hydrogen bonds into chains along the *c* axis. C—H⋯π inter­actions further stabilize the crystal structure.

## Related literature

For the biological properties of thiourea derivatives, see; Sun *et al.* (2006 ▶); Figueiredo *et al.* (2006 ▶). For a related structure, see: Othman *et al.* (2010 ▶); For standard bond lengths, see: Allen *et al.* (1987 ▶).
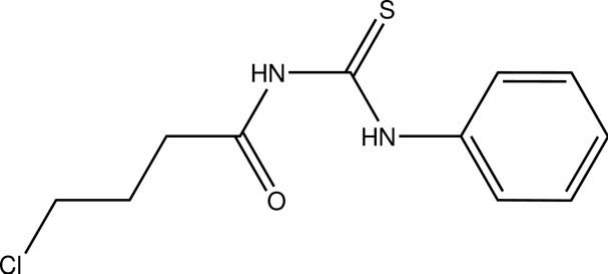


## Experimental

### 

#### Crystal data

C_11_H_13_ClN_2_OS
*M* *_r_* = 256.74
Monoclinic, 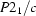

*a* = 14.610 (3) Å
*b* = 10.244 (2) Å
*c* = 18.230 (4) Å
β = 112.408 (4)°
*V* = 2522.5 (9) Å^3^
*Z* = 8
Mo *K*α radiation
μ = 0.45 mm^−1^
*T* = 298 K
0.50 × 0.49 × 0.09 mm

#### Data collection

Bruker SMART APEX CCD area-detector diffractometer
Absorption correction: multi-scan (*SADABS*; Bruker, 2000 ▶) *T*_min_ = 0.807, *T*_max_ = 0.961
14531 measured reflections
4706 independent reflections
3195 reflections with *I* > 2/s(*I*)
*R*_int_ = 0.043

#### Refinement

*R*[*F* ^2^ > 2σ(*F* ^2^)] = 0.048
*wR*(*F* ^2^) = 0.131
*S* = 1.02
4706 reflections
289 parameters
H-atom parameters constrained
Δρ_max_ = 0.48 e Å^−3^
Δρ_min_ = −0.29 e Å^−3^

### 

Data collection: *SMART* (Bruker, 2000 ▶); cell refinement: *SAINT* (Bruker, 2000 ▶); data reduction: *SAINT*; program(s) used to solve structure: *SHELXS97* (Sheldrick, 2008 ▶); program(s) used to refine structure: *SHELXL97* (Sheldrick, 2008 ▶); molecular graphics: *SHELXTL* (Sheldrick, 2008 ▶); software used to prepare material for publication: *SHELXTL*, *PARST* (Nardelli, 1995 ▶) and *PLATON* (Spek, 2009 ▶).

## Supplementary Material

Crystal structure: contains datablocks global, I. DOI: 10.1107/S1600536811001498/hg2783sup1.cif

Structure factors: contains datablocks I. DOI: 10.1107/S1600536811001498/hg2783Isup2.hkl

Additional supplementary materials:  crystallographic information; 3D view; checkCIF report

## Figures and Tables

**Table 1:** Hydrogen-bond geometry (Å, °) *Cg*1 and *Cg*2 are the centroids of the C17–C22 and C6–C11 rings, respectively.

*D*—H⋯*A*	*D*—H	H⋯*A*	*D*⋯*A*	*D*—H⋯*A*
N2—H2⋯O1	0.86	2.04	2.701 (3)	134
N4—H4⋯O2	0.86	2.03	2.692 (3)	133
N1—H1⋯S2^i^	0.86	2.53	3.382 (2)	173
N2—H2⋯O2^ii^	0.86	2.40	3.142 (3)	144
N3—H3⋯S1^iii^	0.86	2.58	3.439 (2)	175
N4—H4⋯O1^ii^	0.86	2.32	3.057 (3)	143
C14—H14*A*⋯S2^iv^	0.97	2.73	3.676 (3)	166
C2—H2*A*⋯*Cg*2^ii^	0.97	2.80	3.419 (4)	123
C13—H13*A*⋯*Cg*1^ii^	0.97	2.83	3.417 (3)	153

Symmetry codes: (i) 
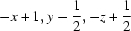
; (ii) 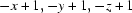; (iii) 
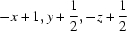
; (iv) 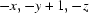.

**Fig. 1.**
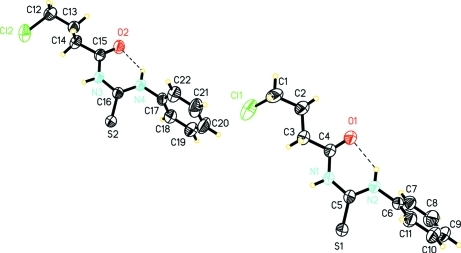
The molecular structure of (I), with displacement ellipsods are drawn at the 50% probability level. the dashed line denote the intramolecular hydrogen bonds.

**Fig. 2.**
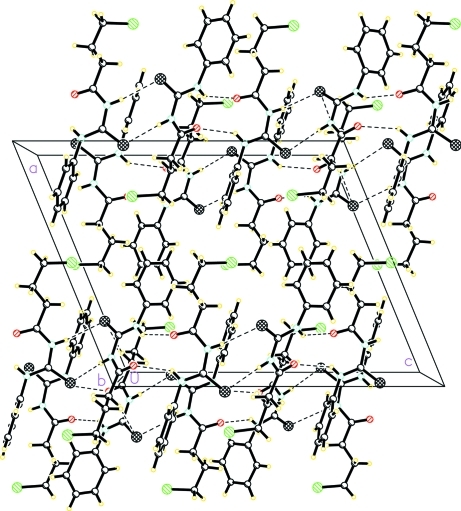
The molecular packing of (I) viewed down the b-axis. The dashed line denote the intermolecular hydrogen bonds.

## supplementary crystallographic information

### Comment

The continuing work on the synthesis of thiourea derivatives is driven by their chemical and biological properties (Sun *et al.* ,2006). Some thiourea derivatives such as *N*-[1-(4*R*)-(4-isopropyl-1-methylcyclohexenyl)]-N'-[2 - (butyl)]thiourea is known to possess anticancer activity (Figueiredo *et al.*, 2006). The title compound (I), is analogous to the previously reported *N*-(3-chloropropionyl)-*N*'-(phenyl)thiourea (Othman *et al.,* 2010) except the terminal chlorine atom is attached at the γ position, 3 (C—C) bonds away from the carbonyl group. The asymmetric unit consists of two independent molecules (Fig.1). Unlike its 3-chloropropionyl analog, the butanoyl group is not planar. However, the thiourea C4/N1/C5/S1/N2/C6, C15/N3/C16/S2/N4/C17 fragments and the benzene rings, (C6—C11) and (C17—C22) are each planar with maximum deviation of 0.059 (2)Å for N3 atom from the least square plane. In each molecule, the benzene ring is vertical to the thiourea fragment with dihedral angle of 72.98 (12)° and 81.47 (14)°, respectively. The bond lengths and angles are in normal ranges (Allen  *et al.*, 1987) and comparable to those in the *N*-(3-chloropropionyl)-*N*'-(phenyl)thiourea. Both molecules maintain the *trans*-*cis* configuration with respect to the position of the carbonoyl and phenyl groups against the thiono C=S bond across their C—N bonds. Such configuration allows the formation of intramolecular hydrogen bonds between the carbonyl oxygen atom and thioamide hydrogen atom, C4—O1···H2- and C15—O2···H4, in both molecules. In the crystal stucture, the molecules are linked by N1—H1···S2, N2—H2···O2, N3—H3···S1, N4—H4···O1 and C14—H14A···S2 intermolecular hydrogen bonds (symmetry codes as in Table 2) forming infinite one-dimensional chains along the *c* axis (Fig.2).The molecule is also stablized by C2—H2A···π and C13—H13A···π with the centroid benzene ring *Cg*2,(C6—C11) and *Cg*1,(C17—C22) respectively (Table 2).

### Experimental

30 ml acetone solution of aniline (1.33 g, 14 mmol) was added into 30 ml acetone containing 4-chlorobutanoy chloride (2.00 g, 14 mmol) and ammonium thiocyanate (1.09 g, 14 mmol). The mixture was refluxed for 2 h. The solution was filtered and left to evaporate at room temperature. The yellowish precipitate obtained after a few days, was washed with water and cold ethanol. The colourless crytals were obtained by recrystallization from ethanol. Yield 90%; m.p 392.3–393.2 K.

### Refinement

All H atoms attached to C and N atoms were fixed geometrically and treated as riding with C—H= 0.93–0.97 Å(aromatic and methylene) and N—H= 0.82 Å(amino) with *U*_iso_(H)=1.2*U*_eq_(C or N).

### Crystal data

**Table:**

C_11_H_13_ClN_2_OS	*F*(000) = 1072
*M**_r_* = 256.74	*D*_x_ = 1.352 Mg m^−^^3^
Monoclinic, *P*2_1_/*c*	Melting point = 392.3–393.2 K
Hall symbol: -P 2ybc	Mo *K*α radiation, λ = 0.71073 Å
*a* = 14.610 (3) Å	Cell parameters from 3372 reflections
*b* = 10.244 (2) Å	θ = 2.3–25.5°
*c* = 18.230 (4) Å	µ = 0.45 mm^−^^1^
β = 112.408 (4)°	*T* = 298 K
*V* = 2522.5 (9) Å^3^	Slab, colourless
*Z* = 8	0.50 × 0.49 × 0.09 mm

### Data collection

**Table:**

Bruker SMART APEX CCD area-detector diffractometer	4706 independent reflections
Radiation source: fine-focus sealed tube	3195 reflections with *I* > 2/s(*I*)
graphite	*R*_int_ = 0.043
Detector resolution: 83.66 pixels mm^-1^	θ_max_ = 25.5°, θ_min_ = 2.3°
ω scans	*h* = −17→16
Absorption correction: multi-scan (*SADABS*; Bruker, 2000)	*k* = −12→11
*T*_min_ = 0.807, *T*_max_ = 0.961	*l* = −22→20
14531 measured reflections	

### Refinement

**Table:**

Refinement on *F*^2^	Primary atom site location: structure-invariant direct methods
Least-squares matrix: full	Secondary atom site location: difference Fourier map
*R*[*F*^2^ > 2σ(*F*^2^)] = 0.048	Hydrogen site location: inferred from neighbouring sites
*wR*(*F*^2^) = 0.131	H-atom parameters constrained
*S* = 1.02	*w* = 1/[σ^2^(*F*_o_^2^) + (0.0595*P*)^2^ + 0.9697*P*] where *P* = (*F*_o_^2^ + 2*F*_c_^2^)/3
4706 reflections	(Δ/σ)_max_ < 0.001
289 parameters	Δρ_max_ = 0.48 e Å^−^^3^
0 restraints	Δρ_min_ = −0.29 e Å^−^^3^

### Special details

**Table:**

Geometry. All e.s.d.'s (except the e.s.d. in the dihedral angle between two l.s. planes) are estimated using the full covariance matrix. The cell e.s.d.'s are taken into account individually in the estimation of e.s.d.'s in distances, angles and torsion angles; correlations between e.s.d.'s in cell parameters are only used when they are defined by crystal symmetry. An approximate (isotropic) treatment of cell e.s.d.'s is used for estimating e.s.d.'s involving l.s. planes.
Refinement. Refinement of *F*^2^ against ALL reflections. The weighted *R*-factor *wR* and goodness of fit *S* are based on *F*^2^, conventional *R*-factors *R* are based on *F*, with *F* set to zero for negative *F*^2^. The threshold expression of *F*^2^ > σ(*F*^2^) is used only for calculating *R*-factors(gt) *etc*. and is not relevant to the choice of reflections for refinement. *R*-factors based on *F*^2^ are statistically about twice as large as those based on *F*, and *R*- factors based on ALL data will be even larger.

### Fractional atomic coordinates and isotropic or equivalent isotropic displacement parameters (Å^2^)

**Table:**

	*x*	*y*	*z*	*U*_iso_*/*U*_eq_	
Cl1	0.49537 (8)	0.21955 (15)	0.48980 (7)	0.1177 (5)	
Cl2	−0.21432 (6)	0.81829 (9)	−0.21567 (5)	0.0734 (3)	
S1	1.01893 (5)	−0.02943 (8)	0.66961 (4)	0.0505 (2)	
S2	0.24956 (5)	0.47421 (8)	0.02400 (4)	0.0479 (2)	
O1	0.79636 (14)	0.1555 (2)	0.75863 (12)	0.0602 (6)	
O2	0.07429 (14)	0.80422 (18)	0.07521 (11)	0.0540 (5)	
N1	0.84965 (15)	0.0558 (2)	0.67035 (12)	0.0433 (5)	
H1	0.8298	0.0332	0.6213	0.052*	
N2	0.98310 (15)	0.0728 (2)	0.79027 (12)	0.0444 (6)	
H2	0.9429	0.1100	0.8080	0.053*	
N3	0.09962 (14)	0.6311 (2)	0.00710 (12)	0.0411 (5)	
H3	0.0729	0.5877	−0.0363	0.049*	
N4	0.23236 (15)	0.6444 (2)	0.12761 (13)	0.0457 (6)	
H4	0.2007	0.7065	0.1393	0.055*	
C1	0.4980 (2)	0.1639 (4)	0.5816 (2)	0.0839 (12)	
H1A	0.4521	0.2154	0.5966	0.101*	
H1B	0.4756	0.0739	0.5760	0.101*	
C2	0.5993 (2)	0.1718 (4)	0.6469 (2)	0.0709 (10)	
H2A	0.6214	0.2619	0.6528	0.085*	
H2B	0.5946	0.1452	0.6965	0.085*	
C3	0.67435 (19)	0.0891 (3)	0.63244 (17)	0.0539 (8)	
H3A	0.6747	0.1104	0.5807	0.065*	
H3B	0.6551	−0.0017	0.6313	0.065*	
C4	0.77790 (19)	0.1056 (3)	0.69406 (16)	0.0449 (7)	
C5	0.94940 (18)	0.0371 (2)	0.71449 (15)	0.0394 (6)	
C6	1.08327 (18)	0.0522 (3)	0.84386 (15)	0.0415 (6)	
C7	1.1446 (2)	0.1570 (3)	0.87086 (18)	0.0564 (8)	
H7	1.1220	0.2408	0.8538	0.068*	
C8	1.2406 (2)	0.1375 (4)	0.9236 (2)	0.0721 (10)	
H8	1.2827	0.2087	0.9423	0.087*	
C9	1.2743 (2)	0.0148 (4)	0.9486 (2)	0.0752 (11)	
H9	1.3394	0.0026	0.9836	0.090*	
C10	1.2127 (2)	−0.0904 (4)	0.9225 (2)	0.0722 (10)	
H10	1.2355	−0.1738	0.9404	0.087*	
C11	1.1162 (2)	−0.0724 (3)	0.86907 (18)	0.0570 (8)	
H11	1.0741	−0.1436	0.8505	0.068*	
C12	−0.2044 (2)	0.8804 (3)	−0.12092 (18)	0.0609 (8)	
H12A	−0.2282	0.9697	−0.1271	0.073*	
H12B	−0.2462	0.8291	−0.1015	0.073*	
C13	−0.09988 (19)	0.8768 (3)	−0.06106 (17)	0.0489 (7)	
H13A	−0.0978	0.9152	−0.0118	0.059*	
H13B	−0.0583	0.9291	−0.0803	0.059*	
C14	−0.05878 (19)	0.7394 (3)	−0.04492 (18)	0.0488 (7)	
H14A	−0.1029	0.6861	−0.0290	0.059*	
H14B	−0.0576	0.7031	−0.0937	0.059*	
C15	0.04357 (19)	0.7319 (3)	0.01824 (16)	0.0418 (6)	
C16	0.19336 (17)	0.5905 (3)	0.05662 (15)	0.0382 (6)	
C17	0.32572 (19)	0.6032 (3)	0.18617 (16)	0.0446 (7)	
C18	0.4123 (2)	0.6520 (3)	0.18414 (19)	0.0616 (8)	
H18	0.4111	0.7105	0.1448	0.074*	
C19	0.5016 (2)	0.6125 (4)	0.2418 (2)	0.0790 (11)	
H19	0.5608	0.6449	0.2413	0.095*	
C20	0.5028 (3)	0.5265 (4)	0.2990 (2)	0.0837 (12)	
H20	0.5629	0.5004	0.3375	0.100*	
C21	0.4155 (3)	0.4778 (4)	0.3003 (2)	0.0798 (11)	
H21	0.4168	0.4189	0.3395	0.096*	
C22	0.3259 (2)	0.5167 (3)	0.2432 (2)	0.0610 (8)	
H22	0.2666	0.4843	0.2437	0.073*	

### Atomic displacement parameters (Å^2^)

**Table:**

	*U*^11^	*U*^22^	*U*^33^	*U*^12^	*U*^13^	*U*^23^
Cl1	0.0732 (7)	0.1601 (12)	0.0867 (8)	0.0387 (7)	−0.0065 (6)	0.0182 (8)
Cl2	0.0749 (6)	0.0718 (6)	0.0519 (5)	−0.0005 (4)	−0.0002 (4)	0.0018 (4)
S1	0.0404 (4)	0.0708 (5)	0.0381 (4)	0.0070 (3)	0.0125 (3)	−0.0009 (3)
S2	0.0404 (4)	0.0594 (5)	0.0391 (4)	0.0100 (3)	0.0101 (3)	−0.0034 (3)
O1	0.0449 (11)	0.0779 (14)	0.0470 (13)	0.0125 (10)	0.0053 (9)	−0.0161 (11)
O2	0.0520 (12)	0.0504 (12)	0.0459 (12)	0.0108 (9)	0.0031 (9)	−0.0066 (10)
N1	0.0356 (12)	0.0581 (14)	0.0299 (11)	0.0061 (10)	0.0054 (9)	−0.0013 (10)
N2	0.0354 (12)	0.0555 (14)	0.0364 (13)	0.0069 (10)	0.0071 (10)	−0.0072 (11)
N3	0.0345 (11)	0.0463 (13)	0.0336 (12)	0.0036 (10)	0.0031 (9)	−0.0036 (10)
N4	0.0383 (12)	0.0511 (13)	0.0381 (13)	0.0097 (10)	0.0039 (10)	−0.0053 (11)
C1	0.0357 (17)	0.118 (3)	0.091 (3)	−0.0003 (19)	0.0159 (17)	−0.035 (2)
C2	0.0382 (16)	0.105 (3)	0.062 (2)	0.0053 (17)	0.0103 (15)	−0.021 (2)
C3	0.0397 (15)	0.068 (2)	0.0451 (17)	0.0045 (14)	0.0066 (13)	−0.0058 (15)
C4	0.0390 (14)	0.0520 (17)	0.0382 (16)	0.0057 (13)	0.0084 (12)	−0.0012 (13)
C5	0.0374 (14)	0.0400 (14)	0.0369 (15)	−0.0003 (11)	0.0098 (11)	0.0044 (12)
C6	0.0345 (14)	0.0529 (17)	0.0332 (14)	0.0032 (12)	0.0086 (11)	−0.0041 (12)
C7	0.0450 (16)	0.0573 (19)	0.060 (2)	−0.0001 (14)	0.0126 (15)	−0.0051 (15)
C8	0.0425 (18)	0.088 (3)	0.073 (2)	−0.0110 (18)	0.0077 (16)	−0.019 (2)
C9	0.0387 (17)	0.114 (3)	0.055 (2)	0.016 (2)	−0.0019 (15)	−0.011 (2)
C10	0.063 (2)	0.079 (2)	0.059 (2)	0.028 (2)	0.0061 (17)	0.0068 (19)
C11	0.0519 (18)	0.0562 (18)	0.0532 (19)	0.0038 (15)	0.0093 (14)	−0.0026 (15)
C12	0.0498 (17)	0.068 (2)	0.056 (2)	0.0158 (15)	0.0109 (15)	0.0062 (16)
C13	0.0448 (15)	0.0491 (17)	0.0470 (17)	0.0048 (13)	0.0110 (13)	−0.0001 (13)
C14	0.0381 (15)	0.0448 (16)	0.0534 (18)	0.0024 (12)	0.0061 (13)	0.0022 (13)
C15	0.0399 (14)	0.0410 (15)	0.0404 (16)	0.0015 (12)	0.0106 (12)	0.0044 (13)
C16	0.0349 (13)	0.0425 (15)	0.0356 (14)	−0.0009 (11)	0.0116 (11)	0.0033 (12)
C17	0.0398 (15)	0.0471 (16)	0.0371 (15)	0.0030 (12)	0.0038 (12)	−0.0082 (13)
C18	0.0460 (17)	0.078 (2)	0.0545 (19)	−0.0024 (16)	0.0128 (15)	−0.0066 (17)
C19	0.0416 (18)	0.112 (3)	0.073 (3)	−0.0035 (19)	0.0094 (17)	−0.021 (2)
C20	0.054 (2)	0.094 (3)	0.073 (3)	0.025 (2)	−0.0097 (19)	−0.011 (2)
C21	0.081 (3)	0.067 (2)	0.064 (2)	0.017 (2)	−0.002 (2)	0.0118 (18)
C22	0.0537 (18)	0.0553 (19)	0.061 (2)	0.0008 (15)	0.0074 (16)	0.0025 (16)

### Geometric parameters (Å, °)

**Table:**

Cl1—C1	1.755 (4)	C7—C8	1.379 (4)
Cl2—C12	1.794 (3)	C7—H7	0.9300
S1—C5	1.673 (3)	C8—C9	1.363 (5)
S2—C16	1.679 (3)	C8—H8	0.9300
O1—C4	1.216 (3)	C9—C10	1.368 (5)
O2—C15	1.214 (3)	C9—H9	0.9300
N1—C4	1.375 (3)	C10—C11	1.387 (4)
N1—C5	1.383 (3)	C10—H10	0.9300
N1—H1	0.8600	C11—H11	0.9300
N2—C5	1.329 (3)	C12—C13	1.501 (4)
N2—C6	1.432 (3)	C12—H12A	0.9700
N2—H2	0.8600	C12—H12B	0.9700
N3—C15	1.381 (3)	C13—C14	1.514 (4)
N3—C16	1.386 (3)	C13—H13A	0.9700
N3—H3	0.8600	C13—H13B	0.9700
N4—C16	1.320 (3)	C14—C15	1.502 (4)
N4—C17	1.438 (3)	C14—H14A	0.9700
N4—H4	0.8600	C14—H14B	0.9700
C1—C2	1.507 (4)	C17—C22	1.366 (4)
C1—H1A	0.9700	C17—C18	1.374 (4)
C1—H1B	0.9700	C18—C19	1.387 (5)
C2—C3	1.487 (4)	C18—H18	0.9300
C2—H2A	0.9700	C19—C20	1.360 (6)
C2—H2B	0.9700	C19—H19	0.9300
C3—C4	1.511 (4)	C20—C21	1.378 (6)
C3—H3A	0.9700	C20—H20	0.9300
C3—H3B	0.9700	C21—C22	1.385 (4)
C6—C7	1.365 (4)	C21—H21	0.9300
C6—C11	1.380 (4)	C22—H22	0.9300
			
C4—N1—C5	129.0 (2)	C9—C10—C11	119.8 (3)
C4—N1—H1	115.5	C9—C10—H10	120.1
C5—N1—H1	115.5	C11—C10—H10	120.1
C5—N2—C6	123.1 (2)	C6—C11—C10	119.3 (3)
C5—N2—H2	118.5	C6—C11—H11	120.4
C6—N2—H2	118.5	C10—C11—H11	120.4
C15—N3—C16	128.5 (2)	C13—C12—Cl2	112.2 (2)
C15—N3—H3	115.7	C13—C12—H12A	109.2
C16—N3—H3	115.7	Cl2—C12—H12A	109.2
C16—N4—C17	122.5 (2)	C13—C12—H12B	109.2
C16—N4—H4	118.7	Cl2—C12—H12B	109.2
C17—N4—H4	118.7	H12A—C12—H12B	107.9
C2—C1—Cl1	113.2 (3)	C12—C13—C14	112.5 (2)
C2—C1—H1A	108.9	C12—C13—H13A	109.1
Cl1—C1—H1A	108.9	C14—C13—H13A	109.1
C2—C1—H1B	108.9	C12—C13—H13B	109.1
Cl1—C1—H1B	108.9	C14—C13—H13B	109.1
H1A—C1—H1B	107.8	H13A—C13—H13B	107.8
C3—C2—C1	113.4 (3)	C15—C14—C13	113.7 (2)
C3—C2—H2A	108.9	C15—C14—H14A	108.8
C1—C2—H2A	108.9	C13—C14—H14A	108.8
C3—C2—H2B	108.9	C15—C14—H14B	108.8
C1—C2—H2B	108.9	C13—C14—H14B	108.8
H2A—C2—H2B	107.7	H14A—C14—H14B	107.7
C2—C3—C4	113.7 (2)	O2—C15—N3	122.4 (2)
C2—C3—H3A	108.8	O2—C15—C14	124.1 (2)
C4—C3—H3A	108.8	N3—C15—C14	113.5 (2)
C2—C3—H3B	108.8	N4—C16—N3	117.6 (2)
C4—C3—H3B	108.8	N4—C16—S2	123.88 (19)
H3A—C3—H3B	107.7	N3—C16—S2	118.51 (19)
O1—C4—N1	123.1 (2)	C22—C17—C18	121.5 (3)
O1—C4—C3	123.8 (2)	C22—C17—N4	118.8 (3)
N1—C4—C3	113.1 (2)	C18—C17—N4	119.7 (3)
N2—C5—N1	117.2 (2)	C17—C18—C19	118.9 (3)
N2—C5—S1	124.53 (19)	C17—C18—H18	120.6
N1—C5—S1	118.25 (19)	C19—C18—H18	120.6
C7—C6—C11	120.7 (3)	C20—C19—C18	120.3 (3)
C7—C6—N2	119.3 (2)	C20—C19—H19	119.9
C11—C6—N2	120.0 (2)	C18—C19—H19	119.9
C6—C7—C8	119.4 (3)	C19—C20—C21	120.4 (3)
C6—C7—H7	120.3	C19—C20—H20	119.8
C8—C7—H7	120.3	C21—C20—H20	119.8
C9—C8—C7	120.5 (3)	C20—C21—C22	120.0 (4)
C9—C8—H8	119.7	C20—C21—H21	120.0
C7—C8—H8	119.7	C22—C21—H21	120.0
C8—C9—C10	120.3 (3)	C17—C22—C21	119.0 (3)
C8—C9—H9	119.9	C17—C22—H22	120.5
C10—C9—H9	119.9	C21—C22—H22	120.5
			
Cl1—C1—C2—C3	−62.3 (4)	Cl2—C12—C13—C14	−61.6 (3)
C1—C2—C3—C4	175.0 (3)	C12—C13—C14—C15	−176.6 (3)
C5—N1—C4—O1	8.8 (5)	C16—N3—C15—O2	3.3 (4)
C5—N1—C4—C3	−170.0 (3)	C16—N3—C15—C14	−174.7 (2)
C2—C3—C4—O1	16.7 (5)	C13—C14—C15—O2	33.0 (4)
C2—C3—C4—N1	−164.5 (3)	C13—C14—C15—N3	−149.0 (2)
C6—N2—C5—N1	176.3 (2)	C17—N4—C16—N3	175.2 (2)
C6—N2—C5—S1	−3.1 (4)	C17—N4—C16—S2	−3.5 (4)
C4—N1—C5—N2	−1.0 (4)	C15—N3—C16—N4	7.5 (4)
C4—N1—C5—S1	178.5 (2)	C15—N3—C16—S2	−173.7 (2)
C5—N2—C6—C7	110.5 (3)	C16—N4—C17—C22	−97.0 (3)
C5—N2—C6—C11	−71.0 (4)	C16—N4—C17—C18	83.5 (3)
C11—C6—C7—C8	0.3 (5)	C22—C17—C18—C19	−0.4 (5)
N2—C6—C7—C8	178.8 (3)	N4—C17—C18—C19	179.1 (3)
C6—C7—C8—C9	0.2 (5)	C17—C18—C19—C20	0.3 (5)
C7—C8—C9—C10	−1.0 (6)	C18—C19—C20—C21	0.0 (6)
C8—C9—C10—C11	1.3 (6)	C19—C20—C21—C22	−0.1 (6)
C7—C6—C11—C10	0.1 (5)	C18—C17—C22—C21	0.2 (5)
N2—C6—C11—C10	−178.5 (3)	N4—C17—C22—C21	−179.3 (3)
C9—C10—C11—C6	−0.8 (5)	C20—C21—C22—C17	0.0 (5)

### Hydrogen-bond geometry (Å, °)

**Table:**

Cg1 and Cg2 are the centroids of the C17–C22 and C6–C11 rings, respectively.

**Table:**

*D*—H···*A*	*D*—H	H···*A*	*D*···*A*	*D*—H···*A*
N2—H2···O1	0.86	2.04	2.701 (3)	134
N4—H4···O2	0.86	2.03	2.692 (3)	133
C3—H3A···Cl1	0.97	2.75	3.194 (3)	109
C14—H14B···Cl2	0.97	2.77	3.180 (3)	106
N1—H1···S2^i^	0.86	2.53	3.382 (2)	173
N2—H2···O2^ii^	0.86	2.40	3.142 (3)	144
N3—H3···S1^iii^	0.86	2.58	3.439 (2)	175
N4—H4···O1^ii^	0.86	2.32	3.057 (3)	143
C14—H14A···S2^iv^	0.97	2.73	3.676 (3)	166
C2—H2A···Cg2^ii^	0.97	2.80	3.419 (4)	123
C13—H13A···Cg1^ii^	0.97	2.83	3.417 (3)	153

Symmetry codes:  (i) −*x*+1, *y*−1/2, −*z*+1/2; (ii) −*x*+1, −*y*+1, −*z*+1; (iii) −*x*+1, *y*+1/2, −*z*+1/2; (iv) −*x*, −*y*+1, −*z*.
